# Erratum: Fission and fusion scenarios for magnetic microswimmer clusters

**DOI:** 10.1038/ncomms14298

**Published:** 2017-01-16

**Authors:** Francisca Guzmán-Lastra, Andreas Kaiser, Hartmut Löwen

Nature Communications 7: Article number: 13519; DOI: 10.1038/ncomms13519 (2016); Published 11 25 2015; Updated 12 17 2017

This Article contains an error in Reference 18 that was introduced during the production process. The correct reference should be:

Steinbach, G. *et al*. Stirrers and movers actuated by oscillating fields. Preprint at arXiv:1607.04733 (2016).

Reference 18 should have been cited in the second paragraph of the ‘Discussion' section as follows: ‘For magnetic dipoles it has been shown that the actuation by an oscillating external magnetic field enables the propulsion of structurally non-deforming chains and rings up to *N*=5 particles^18^.' and ‘The recent experimental developments provide evidence that our predictions about fission and fusion can be verified in granular and colloidal magnetic swimmers^14,18,25^ as well as in magnetotactic bacteria^56–58^.'.

