# Supplementary material for: Fission and fusion scenarios for magnetic microswimmer clusters
Source: Nat Commun. 2016 Nov 22;7:13519. doi: 10.1038/ncomms13519 (PMC5121419; doi:10.1038/ncomms13519)
Supplement: Supplementary Information — Supplementary Figure 1 [file ncomms13519-s1.pdf]

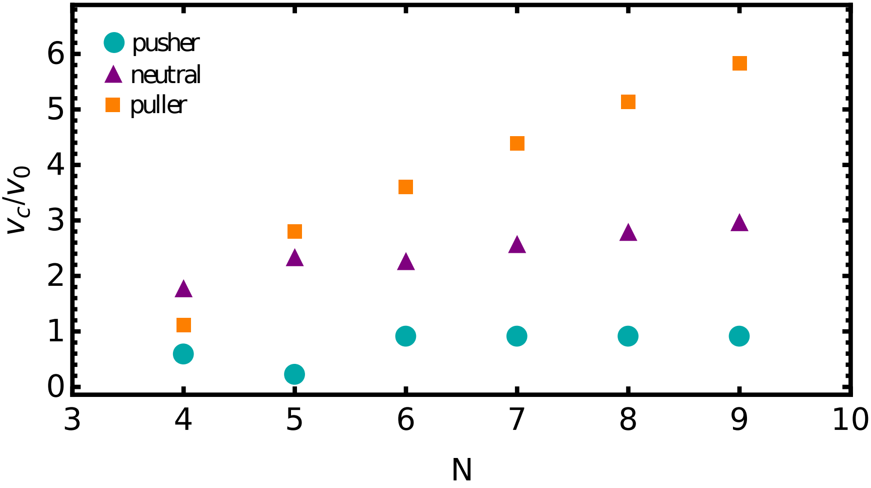

Supplementary Figure 1| Fission state diagram for rings. Emerging state diagram spanned by the reduced velocity and number of particles within a ring-like cluster for all three swimmer types.
